# The Role of Exosomes and Exosomal MicroRNA in Cardiovascular Disease

**DOI:** 10.3389/fcell.2020.616161

**Published:** 2021-01-12

**Authors:** Dongdong Zheng, Ming Huo, Bo Li, Weitie Wang, Hulin Piao, Yong Wang, Zhicheng Zhu, Dan Li, Tiance Wang, Kexiang Liu

**Affiliations:** ^1^Department of Cardiovascular Surgery, The Second Hospital of Jilin University, Changchun, China; ^2^Department of Day Operating Room, The Second Hospital of Jilin University, Changchun, China

**Keywords:** exosome, microRNA, cardiovascular disease, communication, post transcriptional regulation

## Abstract

Exosomes are small vesicles (30–150 nm in diameter) enclosed by a lipid membrane bilayer, secreted by most cells in the body. They carry various molecules, including proteins, lipids, mRNA, and other RNA species, such as long non-coding RNA, circular RNA, and microRNA (miRNA). miRNAs are the most numerous cargo molecules in the exosome. They are endogenous non-coding RNA molecules, approximately 19–22-nt-long, and important regulators of protein biosynthesis. Exosomes can be taken up by neighboring or distant cells, where they play a role in post-transcriptional regulation of gene expression by targeting mRNA. Exosomal miRNAs have diverse functions, such as participation in inflammatory reactions, cell migration, proliferation, apoptosis, autophagy, and epithelial–mesenchymal transition. There is increasing evidence that exosomal miRNAs play an important role in cardiovascular health. Exosomal miRNAs are widely involved in the occurrence and development of cardiovascular diseases, such as atherosclerosis, acute coronary syndrome, heart failure (HF), myocardial ischemia reperfusion injury, and pulmonary hypertension. In this review, we present a systematic overview of the research progress into the role of exosomal miRNAs in cardiovascular diseases, and present new ideas for the diagnosis and treatment of cardiovascular diseases.

## Introduction

Exosomes were originally identified in differentiated reticulocytes by Pan et al. (1985). The authors demonstrated that transferrin and many membrane-associated proteins are secreted from the cell in a vesicle during reticulocyte maturation, and they defined this vesicle as an exosome. At first, exosomes were thought to be involved in waste excretion from the cell, and they remained largely unexplored in the following decade. This changed in 1996, when Raposo et al. (1996) showed that exosomes from human and mouse B lymphocytes induce the response of antigen-specific major histocompatibility complex (MHC) II-restricted T lymphocytes, which indicated that these vesicles play an antigen-presenting role. Since then, exosomes have received increasing attention from the researchers, and it has been suggested that they may be a means of information transmission between cells. With the continuing research, diverse functions of exosomes have been discovered, such as participation in the immune response, antigen presentation, tumor cell migration and proliferation, apoptosis, autophagy, and so on (Bobrie et al., 2011; Hannafon and Ding, 2013; Robbins et al., 2016; Spees et al., 2016; Harada et al., 2017; Papandreou and Tavernarakis, 2017; Lindenbergh and Stoorvogel, 2018; Zeng et al., 2018; Zhang S. et al., 2018). In recent years, a large number of studies have shown that miRNAs use exosomes as carriers to achieve cell-to-cell communication (Asgarpour et al., 2020). Specifically, exosome miRNAs are secreted by donor cells in the form of paracrine or remote secretion, and then taken up by donor cells. The exosome miRNAs enter the recipient cells, and form a RISC complex by targeting the mRNA 3′UTR and AGO2 protein in the recipient cell to degrade the mRNA of the target gene or inhibit the process of mRNA translation into protein. For example, Barile et al. (2014) showed that among exosome miRNAs secreted by cardiac progenitor cells (CPCs), the miRNAs with the highest content include miR-210, miR-132, and miR-146a-3p. Among them, after the exosome miR-210 is taken up by cardiomyocytes, it can down-regulate the expression of ephrin A3 and PTP1b to inhibit cardiomyocyte apoptosis. After exosome miR-132 is taken up by umbilical vein endothelial cells, the expression of RabGAP-P120 can be down-regulated by targeting to promote endothelial cell formation into tubes (Barile et al., 2014). Besides, as exosomes can protect miRNAs from harsh environments, exosomes miRNAs can stably exist in human plasma and body fluids. Therefore, the structural stability of exosome miRNAs makes it a non-invasive biomarker for disease prognosis and disease monitoring, such as cervical cancer and malignant glioma (Ghaemmaghami et al., 2020; Nahand et al., 2020). With further research, exosome miRNAs can be used as biomarkers for the diagnosis and prognosis of acute cardiovascular events. In this review, we present an overview of the current state of the knowledge on exosome biogenesis and function, with a specific focus on exosomal microRNA (miRNA) involvement in cardiovascular diseases, and the potential of exosomal miRNAs as novel diagnosis and treatment tools.

## Overview of Exosome Formation

### Exosome Biogenesis

**FIGURE 1:**
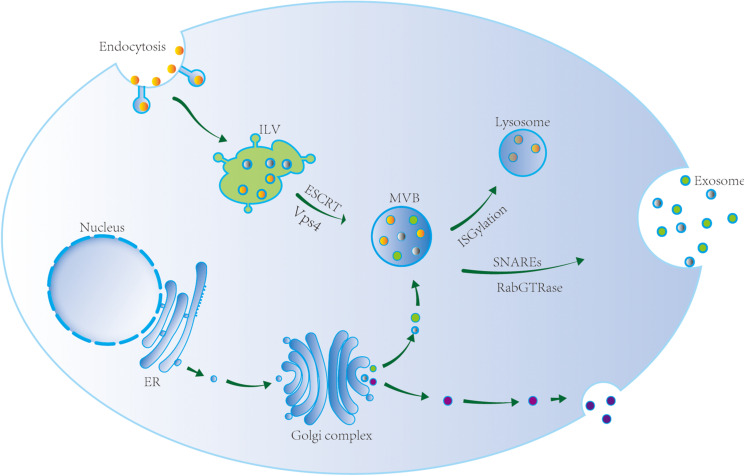
Exosome biogenesis, sorting, and trafficking. Exosomes originate from the endosome system. The endosome membrane invaginates and sprouts to form intraluminal vesicle (ILV), the early endosome. ILV matures to form multivesicular body (MVB) via ESCRT and Vsp4, i.e., the late endosome. MVB reach lysosome after ISGylation and its contents are degraded, or is transported to the cell membrane to release exosomes via SNAREs and RabGTPase.

Exosomes originate from the endosome system (Figure 1). The endosome membrane invaginates and sprouts to form intraluminal vesicles (ILVs), which are early endosomes. ILVs further mature to form multivesicular bodies (MVBs), which are late endosomes (Hessvik and Llorente, 2018; Gurunathan et al., 2019). The endosomal-sorting complex required for transport (ESCRT) plays an important role in MVB formation. ESCRT complexes include ESCRT-0, ESCRT-I, ESCRT-II, ESCRT-III, and Vps4 (Schmidt and Teis, 2012). In addition, MVBs also form via an ESCRT-independent pathway (Marsh and van Meer, 2008). Stuffers et al. (2009) observed that ILVs form MVBs even after ESCRT inhibition, indicating the existence of such an ESCRT-independent mechanism for MVB formation. Transmembrane proteins that are abundant in exosomes are involved in this process. van Niel et al. (2011) knocked out the expression of CD63 in HEK293 cells and observed a significant reduction in MVB production. This indicated that the transmembrane protein CD69 plays an important role in mediating the formation of MVBs via an ESCRT-independent mechanism. Further, Chairoungdua et al. (2010) demonstrated that CD9 and CD82 also play an important role in the ESCRT-independent mechanism. The authors showed that CD9 and CD82 promote exosome β-catenin release.

According to current research, the sorting mechanism depending on the ESCRT-dependent pathway may be involved in lysosomal degradation of proteins in ILVs, while the sorting mechanism depending on the ESCRT-independent pathway may be involved in ILV secretion. However, the process of MVB formation is likely to be much more complicated, and may depend on the cell type, cargo, and stimulation conditions. In addition, the transformation between two mechanisms mentioned above is still unclear.

### MVB Transport

Multivesicular bodies can reach lysosomes, where their contents are then degraded or transported to the plasma membrane to release exosomes. MVB transport to the cell membrane and fusion with the cell membrane depends on microtubules, actin cytoskeleton, t-soluble *N*-ethylmaleimide-sensitive factor attachment protein receptor (SNARE), v-SNARE, and Rab GTPases. Sinha et al. (2016) showed that the actin skeleton regulator cortactin promotes exosome secretion. As revealed by live-cell imaging, cortactin controls MVB transport and cell membrane docking. Cortactin, Rab27, and coronin 1b cooperatively control the stability of the docking site between actin MVB and the cell membrane, and exosome secretion (Sinha et al., 2016).

The exact mechanism of MVB fusion with the plasma membrane is not clear. The known proteins involved in the fusion are SNAREs, Rabs, and Ras GTPase. SNAREs play an important role in promoting the fusion of MVBs and target membranes. The SNARE family includes v-SNARE and t-SNARE (Bonifacino and Glick, 2004). Fader et al. (2009) reported that v-SNARE vesicle-associated membrane protein 7 is necessary for exosome release from the human leukemia cell line K562. However, little is known about the mechanism of fusion between MVBs and lysosomes. Villarroya-Beltri et al. (2016) proposed that ISGylation controls the fate of MVBs. ISGylation is a ubiquitin-like protein modification process. After induction of ISGylation, the number of MVBs in cells dramatically decreases, and the secretion of exosomes also notably decreases. Indeed, ISGylation triggers the fusion of MVBs and lysosomes, leading to the aggregation and degradation of MVB proteins in lysosomes. Inhibition of lysosome function or autophagy can restore exosome secretion. These observations indicate that ISGylation is a new ubiquitination-like modification that controls the secretion of exosomes.

## Exosomal miRNAs

### Biogenesis of miRNAs

**FIGURE 2:**
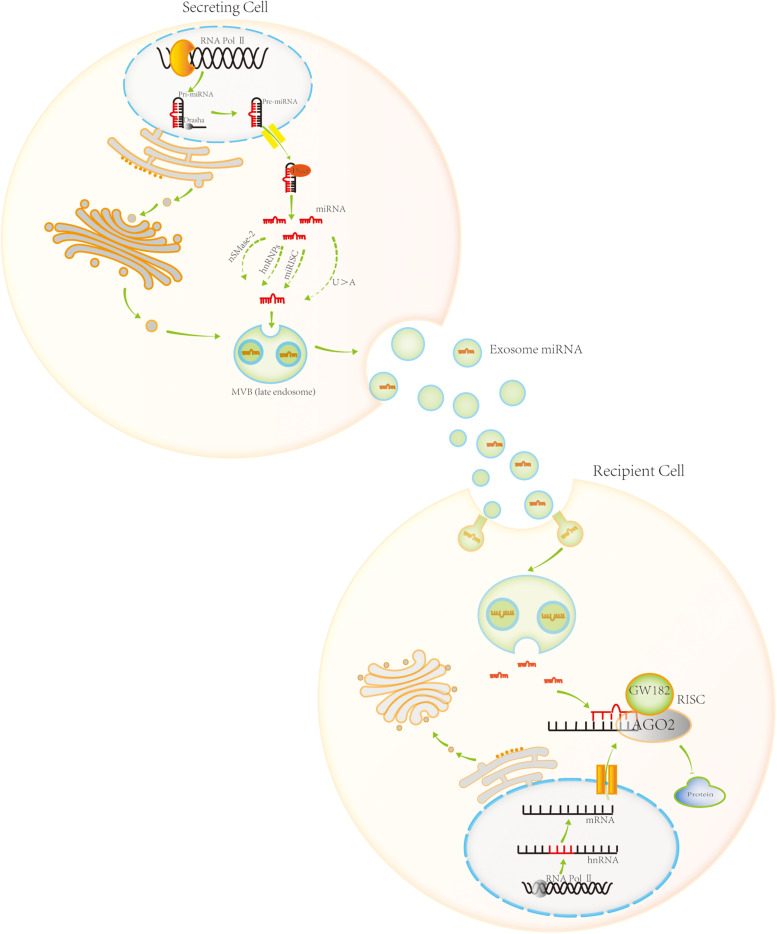
Exosomal miRNA biogenesis, sorting, trafficking, and biological function. Most miRNAs are encoded by their own genes. RNA polymerase II transcribes these genes, producing pri-miRNA. Pri-miRNA is processed in the nucleus by Drasha, generating pre-miRNA. Pre-miRNA is then transported to the cytoplasm by Exportin 5. It is further processed in the cytoplasm by Dicer, generating mature miRNA. Four sorting pathways exist for miRNA entry into the exosome, namely, the sphingomyelinase 2-dependent pathway, hnRNP-dependent pathway, miRNA-induced silencing complex (miRISC) associated pathway, and sequence-dependent pathway involving the 3′-end of miRNA. Exosomal miRNA plays a role in post-transcriptional gene regulation upon uptake by neighboring or distant cell. It enters the target cell and combines with the target mRNA molecule based on partial sequence complementarity. miRNAs inhibit protein synthesis by forming miRISC, a complex with specific proteins. AGO2 and GW182 are the core components of miRISC.

MicroRNAs are endogenous non-coding RNAs that are approximately 19–22-nt and play an important role in the regulation of protein biosynthesis. There are two main sources of miRNAs in eukaryotic cells. Most miRNAs are encoded by their own genes, transcribed by the RNA polymerase II, and processed to produce miRNAs. However, a small number of miRNA sequences are encoded within other RNA molecules. Following transcription of the corresponding RNA molecules, miRNAs can be produced via specific processing mechanisms, such as long non-coding RNA (lncRNA) (Figure 2).

In most cases, specific miRNA transcripts are generated by RNA polymerase II, which is a classic method of miRNA biosynthesis (Karube et al., 2005; Lin and Gregory, 2015; Wojciechowska et al., 2017; Lu and Rothenberg, 2018). Transcription produces pri-miRNA, the primary precursor of miRNA. Pri-miRNA is modified post-transcriptionally, with a cap structure added at the 5′-end and a polyA tail added at the 3′-end. The pri-miRNA is usually several thousand nucleotides long, and can form a stem-loop structure, with the mature miRNA sequence in the stem portion (the RNA fragments forming the stem are not entirely complementary). Pri-miRNA is processed in the nucleus by the RNase III family protein Drasha. In mammals, Drasha combines with the double-stranded RNA-binding protein DiGeorge Syndrome critical region 8 homolog (DGCR8) to form a Drasha–DGCR8 complex. The complex processes pri-miRNA into a miRNA precursor of approximately 70-nt, the pre-miRNA. Pre-miRNA has a hairpin structure (Lee et al., 2003; Gregory et al., 2004; Han et al., 2004; Merritt et al., 2008; Walz et al., 2015). It is transported to the cytoplasm by the export protein Exportin 5 (Yi et al., 2003). In the cytoplasm, Dicer, another member of the RNase III family, processes the pre-miRNA. Dicer binds to the double-stranded RNA-binding protein TRBP to form a complex that cleaves the pre-miRNA. The loop structure at the end of the double strand is cut off, producing a miRNA/miRNA^∗^ double-stranded molecule of approximately 20 bp (Karube et al., 2005). After processing, if the two strands of short-chain RNA molecules are not completely complementary, they bind AGO 1 protein to form miRNA–RNA-induced silencing complex (miRISC). If the two strands of short-chain RNA molecules are complementary, they bind AGO2 protein to form another miRISC. Generally, miRNA^∗^ on one strand of the double-stranded RNA molecule is released and degraded, whereas miRNA on the other strand remains in the RISC complex. However, miRNA^∗^ is not always a byproduct of processing, and sometimes it is loaded into the RISC complex to play the role of miRNA (Jonas and Izaurralde, 2015; Michlewski and Cáceres, 2019).

Although most miRNAs are transcribed from specific miRNA genes, this is not the only way in which miRNAs are generated. Pre-miRNA sequences can also be found in introns of protein-coding genes or in other non-coding RNAs. Intron sequences of some protein-coding genes contain miRNA precursors, and transcribed RNA can form pri-miRNA containing a stem-loop structure. Then, the Drosha–DGCR8 complex cuts pri-miRNA from the bottom of the stem-loop and releases pre-miRNA of approximately 70 nt. Some miRNA sequences are found in very short intron sequences. In this case, miRNA is produced from the intron sequences of protein-coding genes post-transcription. Specifically, miRNA is produced by an intron splicing mechanism, which does not require the participation of the Drosha–DGCR8 complex. The follow-up processing of pre-miRNAs from introns of protein-coding genes is the same as that for pre-miRNA from miRNA genes, and pre-miRNA is transported to the cytoplasm by Exportin 5. In the cytoplasm, it is further processed by the Dicer–TRBP complex to form miRNA/miRNA^∗^ double-strands of approximately 20 bp.

In addition, miRNAs can be generated from long non-coding (lnc) RNA. One of the functions of lncRNA may be to act as a small-RNA precursor (less than 200-nt–long). Some miRNAs are produced by lncRNA processing, usually after cutting of the lncRNA chain by the Drosha and Dicer enzymes (Achkar et al., 2016).

### Exosomal miRNA Sorting

Exosomes contain a variety of molecules, including proteins, lipids, DNA, mRNA, miRNA, lncRNA, and circular RNA. Among them, exosomal miRNAs are the most numerous exosomal cargo molecules. They are involved in post-transcriptional regulation of gene expression, and have attracted much attention. Although the underlying mechanisms are not entirely clear, four possible pathways for miRNA sorting into exosomes have been identified (Figure 2). The first one is the sphingomyelinase 2-dependent pathway. Kosaka et al. (2013) demonstrated that sphingomyelinase 2 promotes the secretion of exosomal miRNA. The second pathway is the heterogeneous nuclear ribonucleoprotein (hnRNP)-dependent pathway. Villarroya-Beltri et al. (2013) showed that hnRNPA2B1 controls exosomal miRNAs entry to the exosomes. The third pathway is the miRISC-associated pathway. Therein, mature miRNA interacts with the assembly proteins to form RISC complexes. The miRISC complex is composed of miRNA, mRNA inhibited by the miRNA, AGO2, and glycine-tryptophan protein of 182 Da (GW182). AGO2 preferentially binds to U or A at the end of miRNA, which plays an important role in mediating the formation of the miRNA:mRNA complex, and then degrading the mRNA molecule or blocking its translation. Frank et al. (2010), Guduric-Fuchs et al. (2012) observed that knocking out AGO2 in HEK293T cells results in a significant reduction in the exosomal miRNA release from the cells, such as exosomal miR-142-3p, miR-150, and miR-451. The final, fourth, pathway is the sequence-dependent pathway involving the 3′-end of miRNA. Lee et al. (2009), Koppers-Lalic et al. (2014) analyzed small-RNA profiles of human B cells and their secreted exosomes by RNA sequencing. Comprehensive bioinformatics and statistical analyses revealed that miRNAs are not randomly distributed in the B cells and their secreted exosomes. Specifically, miRNAs with an adenylated 3′-end are relatively enriched in the cells, while those with an uridylated 3′-end are relatively enriched in the exosomes. This indicates that the post-transcriptional modification of the 3′-end of miRNA plays an important role in guiding miRNAs into the exosome.

### Function of Exosomal miRNAs

Once they are taken up by neighboring or distant cell, exosomal miRNAs play an important role in post-transcriptional regulation of gene expression. Exosomal miRNAs enter the target cell and combine with the mRNA of the target gene based on partial sequence complementarity. Seven nucleotides (nucleotides 2–8) at the 5′-end of miRNA comprise the seed region, which is key for mRNA recognition. The miRNA binding to a target mRNA regulates protein synthesis by forming miRISC.

Argonaute protein is the core component of RISC and directly binds miRNA. There are four AGO proteins in mammalian cells, AGO1–AGO4. The majority of miRNAs bind AGO1, while some bind AGO2. MiRISC formed by the combination of AGO1 and miRNA mainly inhibits protein translation, while miRISC formed by the combination of AGO2 and miRNA can shear target mRNA, resulting in gene expression silencing. GW182 protein is another important component of miRISC. They are recruited to miRNA targets through direct interactions with AGO2 proteins and promote target mRNA silencing.

The function of miRISC is to guide miRNA to bind to target mRNA. The complex regulates the translation of target mRNA in different ways. For example, Zeng et al. (2018) showed that in colorectal cancer, exosomal miR-25-3p is transferred from colorectal cancer cells to endothelial cells, where it regulates the expression of VEGFR2, ZO-1, occludin, and claudin5 by targeting KLF2 and KLF4, thus increasing vascular permeability and promoting angiogenesis. As another example, Hsu et al. (2017) demonstrated that exosomal miR-23 produced by lung cancer cells under hypoxia can promote angiogenesis and increase vascular permeability compact protein via ZO-1. In addition, Fabbri et al. (2012) observed that exosomal miR-21 and miR-29a secreted by tumor cells play a role in immune response, via another mechanism, i.e., by binding with toll-like receptors (TLRs) on immune cells. The binding triggers TLR-mediated inflammatory reactions, ultimately promoting tumor cell proliferation and metastasis. Additional studies are needed to delineate this function of exosomal miRNA.

## Cardiovascular Diseases and Exosomal miRNAs

### Atherosclerosis and Exosomal miRNAs

Atherosclerosis is one of the most common vascular diseases, and the root cause of myocardial infarction and stroke. It is usually characterized by endothelial cell injury, aggregation of inflammatory cells and vascular smooth muscle cells (VSMCs), and deposition of extracellular lipids and fibrous tissue. Endothelial dysfunction is considered an early marker of atherosclerosis, which precedes the evidence of angiography or ultrasound of atherosclerotic plaques. Generally, endothelial cells are highly adaptable to injury stimuli, such as inflammatory stimuli and hyperlipidemia. Damaged endothelial cells are rapidly replaced by proliferating resident endothelial cells. However, as the injury stimulus persists, the endothelial cells undergo apoptosis and necrosis. Atherosclerosis occurs preferentially during laminar flow disorder, leading to endothelial cell injury, followed by chronic inflammation and VSMC proliferation. Under certain pathological conditions, the vascular wall cells are activated and highly proliferate, which resulted in the thickening and hardening of the arterial wall. Activated VSMCs play an important role in the progression of atherosclerosis. Therefore, preventing endothelial cell and VSMC dysfunction may reduce the risk of atherosclerosis-associated diseases, and provide a new treatment method.

Exosomal miRNAs play an important role in atherosclerosis and mediate intercellular communication (Libby et al., 2011; Hergenreider et al., 2012; Lovren et al., 2012; Gorenne et al., 2013; Hulsmans and Holvoet, 2013; Verweij et al., 2015; Blaser and Aikawa, 2019). Specifically, Hergenreider et al. (2012; Lovren et al., 2012) showed that exosomes secreted by human umbilical vein endothelial cells mediated by KLF2 stimulated by shear force are enriched in miR-143/145, which control the expression of target genes in co-cultured smooth muscle cells. Exosomes produced by endothelial cells overexpressing KLF2 reduce the formation of aortic sclerosis injury in ApoE(–/–) mouse. Further, according to an increasing number of studies, exosomal miRNAs of mesenchymal stem cells (MSCs) exert anti-atherosclerotic effects. For instance, Li J. et al. (2019) fed ApoE(–/–) mice high-fat diet and used intravenous injection to administer exosomes from MSCs for 12 weeks in the mouse model. Following tail-vein injection, exosomes secreted by MSCs migrated to atherosclerotic plaques and resided near the macrophages. MSC exosomes reduced the atherosclerotic plaque area in these mice, greatly reduced the plaque infiltration by macrophages, and induced the polarization of macrophages to M2 type. In the same study, the enrichment of miR-let7 family in the MSC exosomes was increased. Endogenous miR-let7 levels increased in the aortic root of ApoE(–/–) mice, and the miR-let7 levels were further upregulated by MSC exosome treatment. In addition, knocking down miR-let7 expression in U937 cells inhibited cell migration and M2 polarization. Another study revealed that miR-let7 secreted by MSCs inhibits macrophage infiltration through the IGFZBP1/PTEN pathway and promotes the polarization of M2 macrophages through the HMGA2/NF-κB pathway.

The interaction between endothelial cells and macrophages mediated by exosomes plays an important role in the pathogenesis of atherosclerosis (Nguyen et al., 2018). Xing et al. (2020) showed that exosomal miR-342-5p from adipose-derived MSCs exerts an anti-atherosclerotic effect on endothelial cells. Further, He et al. (2018) showed that the treatment of endothelial cells with oxidized low-density lipoprotein or overexpression of KLF2 results in enhanced exosomal miR-155 levels in endothelial cells. Exosomal miR-155 can polarize macrophages to M2 cells, which inhibit inflammatory reactions. In addition, Chang et al. (2019; Loyer et al., 2014) reported that the release of exosomal miR-92a upon endothelial cell injury increases during atherosclerosis. Exosomal miR-92a is taken up by macrophages in the local microenvironment, and activates macrophages by a targeted regulation of KLF4 to participate in the formation of atherosclerotic plaque. Zheng et al. (2017) showed that overexpression of KLF5 in smooth muscle cells promotes the expression of miR-155 and acts on adjacent endothelial cells, thus destroying the tight connection between endothelial cells and the integrity of endothelial barrier.

According to some recent studies, macrophages and VSMCs communicate with each other via exosomal miRNA during the onset of atherosclerosis. For example, Zhu et al. (2019) observed that stimulating macrophages with nicotine stimulates the release of exosomal miR-21-3p from macrophages. Exosomal miR-21-3p is taken up by neighboring VSMCs, promoting their proliferation and migration via targeted regulation of PTEN. Further, Liu et al. (2020) showed that oxidized low-density lipoprotein (ox-LDL) stimulates macrophages, which then secrete exosomal miR-106a-3p to promote VSMC proliferation and inhibit VSMC apoptosis.

During the development of atherosclerosis, platelets can communicate with endothelial cells via exosomes as well. For example, Li et al. (2017) showed that the levels of miR-223, miR-339, and miR-21 in the exosomes released by platelets increase after thrombin activation of platelets. Exosomal miR-223 can induce the expression of ICAM-1 stimulated by TNF-α in endothelial cells, thus inhibiting inflammatory reaction.

In addition, dendritic cells and endothelial cells can also communicate with each other via exosomes. For example, Zhong et al. (2019) demonstrated that exosomal miR-146a secreted by dendritic cells is taken up by endothelial cells, where it regulates the inflammatory response by inhibiting IRAK-1.

### Acute Coronary Syndrome (ACS) and Exosomal miRNAs

Acute coronary syndrome is a group of clinical syndromes whose pathological basis is the rupture or invasion of coronary atherosclerotic plaque, followed by complete or incomplete occlusive thrombosis, including ST-segment elevation myocardial infarction, acute non-ST segment elevation myocardial infarction, and unstable angina pectoris (UA). ACS is a common and serious coronary heart disease. ACS patients frequently experience paroxysmal chest pain, chest tightness, and other symptoms. ACS often leads to arrhythmia, HF, and even sudden death, hence seriously affecting the individual’s quality of life. Receiving an appropriate treatment in a timely manner can greatly reduce the mortality and ACS complications, and improve the prognosis of patients.

According to recent studies, exosomal miRNAs play an important role in ACS. Generally speaking, it is anticipated that exosomal miRNAs will become an important tool in diagnosing and treating ACS (Ong et al., 2014; Sahoo and Losordo, 2014; Khan et al., 2015; Mathiasen et al., 2015; Saparov et al., 2017). For example, Bi et al. (2015) reported that exosomal miR-208a levels in the serum of ACS patients are significantly higher than those in healthy individuals. In that study, the average age of the ACS patient population analyzed (*n* = 500) was 62.35 ± 9.70 years, with 300 patients with low miR-208a levels and 200 patients with high miR-208a levels. The high-level miR-208a group was older, with higher Killip grade, and higher peak value of CK-MB than those in the low-level miR-208a group. After 1 year of follow-up, 32 patients died, with 10 cases in the low-level miR-208a group (mortality rate of 3.3%) and 22 patients in the high-level miR-208a group (mortality rate of 11.0%). According to the Kaplan–Meier survival analysis, the 1-year survival rate of patients with high miR-208a levels decreased significantly compared with low miR-208a level group. Further, Ling et al. (2020b) showed that the levels of miR-126 and miR-21 in UA and acute myocardial infarction (AMI) patients are significantly higher than those in healthy controls. According to the Gensini score, circulating exosomal miR-126 levels are positively correlated with coronary stenosis in patients with UA and AMI. Hence, miR-181a could be used as a new biomarker for diagnosing AMI.

Ling et al. (2020a) explored the exosomal miR-122-5p as a biomarker of UA and AMI, and asked whether its levels were positively correlated with the degree of coronary artery stenosis. Indeed, the exosomal miR-122-5p levels in the serum of patients with UA and AMI were significantly higher than those in the control group. Receiver operating characteristic (ROC) curve analysis revealed that the serum exosomal miR-122-5p levels could be used as a diagnostic marker for AMI and UA. In addition, according to the Gensini score, serum levels of miR-122-5p were positively correlated with the degree of stenosis in UA patients. Therefore, increased levels of exosomal miR-122-5p in the serum can be used to predict the degree of coronary artery stenosis.

In addition, Xue et al. (2019) reported that miR-17-5p, miR-126-5p, and miR-145-3p levels in the serum of patients with AMI were significantly elevated compared with health group. Based on ROC curve analysis, the miR-17-5p, miR-126-5p, and miR-145-3p levels have a high diagnostic value for AMI. Similar, Faccini et al. (2017) reported that the levels of let-7c, miR-145c, and miR-155 in CAD patients were significantly lower than those in the control group. Accordingly, ROC curve analysis indicated that let-7c, miR-145c, or miR-155 levels were effective markers for CAD detection.

It has been also reported that, following AMI, damaged myocardial cells and mononuclear cells in the bone marrow can communicate with each other remotely via exosomal miRNA. For example, Saha et al. (2019) showed that injured myocardial cells release exosomal miR-1, exosomal miR-208, and exosome miR-499 after AMI, which then enter the bone marrow to down-regulate the expression CXCR4 and increase the number of circulating monocytes.

Myocardial infarction is caused by myocardial ischemia, which is the main cause of morbidity and sudden death worldwide. In the process of myocardial infarction and subsequent HF, myocardial cells undergo remodeling, such as myocardial cell apoptosis, extracellular matrix protein increase, fibrosis, myocardial cell hypertrophy, and others. Therefore, the repair of myocardial cells and reversing myocardial remodeling are the key to treating HF. MSCs are considered to be a potential source of tissue-specific cells because of their pluripotency, low immunogenicity, availability, and expandability, and have been used in clinical trials to treat refractory diseases. Indeed, MSC transplantation can effectively reduce the left ventricular-end systolic volume and improve left ventricular ejection fraction in patients with severe ischemic HF. In-depth studies revealed that MSCs repair damaged cardiomyocytes using exosomal miRNAs (Woodall and Gustafsson, 2018). For example, Zhu et al. (2018) showed that following hypoxia-induced MSCs, the release of exosomal miR-125b from MSCs increases. Exosomal miR-125b derived from MSCs effectively reduces the myocardial infarction area and promotes the repair of myocardial cells. In addition, research from the Luther group revealed that exosomal miR-21a-5p secreted by MSCs plays a role in myocardial protection by down-regulating the expression of PDCD4, PTEN, Peli1, and FasL in cardiomyocytes (Wang et al., 2017; Luther et al., 2018). Similarly, Li Y. et al. (2019) demonstrated that exosomal miR-301 derived from bone marrow MSCs (BMSCs) reduces the area of myocardial infarction and improves autophagy of myocardial cells. Further, Peng et al. (2020) showed that miR-25-3p secreted by MSCs reduces myocardial infarction area by targeting EZH2; Wen et al. (2020) showed that exosomal miR-144 derived from MSCs targets the regulation of the PTEN/AKT pathway, thus improving the apoptosis of cardiomyocytes under hypoxia. Geng et al. (2020) showed that miR-143, which is derived from serum exosomes, promotes myocardial angiogenesis through the IGF-IR/NO pathway. Collectively, based on the above, exosomal miRNA is expected to become a new method for the diagnosis and treatment of ACS.

### Ischemia Reperfusion Injury and Exosomal miRNAs

Myocardial ischemia reperfusion injury (MIRI) refers to the phenomenon in which the blood supply of the myocardial tissue is restored after a period of its interruption, but the injury from myocardial tissue is aggravated. The pathogenesis of MIRI has not been fully elucidated but it includes oxygen free-radical injury, intracellular calcium overload, and inflammatory injury (Ibáñez et al., 2015; Tibaut et al., 2017).

Recent studies have shown that exosomal miRNAs play an important role in MIRI. For example, Wang et al. (2019) showed that exosomal miR-126 reduces apoptosis in neonatal rat cardiomyocytes treated with H_2_O_2_ and CoCl_2_, and improves the cell survival rate. In addition, exosomal miR-126 significantly improves cardiac function. This indicates that exosomal miR-126 can reduce MIRI by targeting and regulating ERRFI. Luo et al. (2019) showed that hypoxia and reoxygenation (H/R) significantly increase exosome secretion by cardiac fibroblasts (CFs) in a Transwell co-culture system, thus protecting H9C2 cells from H/R damage. Further analysis revealed that exosomal miR-423-3p secreted by CFs improves the viability of H2C9 cells and reduces apoptosis by targeting RAP2C.

BMSCs are fibroblast-like pluripotent adult stem cells that exist in the bone marrow microenvironment. Injecting exosomes secreted by BMSCs into the infarcted area can significantly reduce the infarcted area and repair the function of myocardial cells after MIRI. According to recent studies, exosomes derived from BMSCs can promote the survival of cardiomyocytes and inhibit myocardial cell apoptosis after MIRI (Lu et al., 2015; Liu et al., 2017; Jiang et al., 2018). For example, Zhu et al. (2018) showed that exosomal miR-125b derived from BMSCs reduces MIRI by targeting SIRT7. Further, Sun et al. (2019) demonstrated that exosomes secreted by BMSCs induce the proliferation of H9C2 cells and inhibit the apoptosis of H9C2 cells in the H/R model, which indicates that these exosomes exert a protective effect against myocardial cell injury caused by H/R. In addition, exosomal miR-486-5p derived from BMSCs can repair myocardial injury caused by MIRI via PTEN/PI3K/Akt pathway. Zhao et al. (2019) showed that myocardial injection of BMSCs after MIRI effectively reduces the myocardial infarction area and alleviates the inflammatory reaction at the heart tissue and serum levels. The authors also found that miR-182, an exosome derived from BMSCs, promotes the polarization of M2 macrophages via TLR4, inhibits the inflammatory reaction, and plays a role in myocardial protection. Finally, it demonstrated that MSC-Exo reduces MIRI in mice via exosomal miR-182 that modifies the polarization status of macrophages. Therefore, based on the above, exosomal miRNAs derived from CFs and BMSCs are expected to become a new method for alleviating MIRI. In addition, recent studies have shown that exosomes derived from CDCs or cardiac-resident progenitor cells (CPCs) have a protective effect on cardiomyocytes. For example, Cambier et al. (2017) showed that exosomes Y RNA fragment (YF1) secreted by CDCs can reduce the area of myocardial infarction after MIRI. Ciullo et al. (2019) demonstrated that intramyocardial injection of exosomes derived from CPCs significantly reduced scars after myocardial infarction in rats and improved cardiac function. Barile et al. (2018) further compared the protective effects of exosomes derived from CPC and BMC on the myocardium. This study shows that exosomes derived from CPCs are more effective in reducing the size of scars and improving heart function after coronary artery occlusion in rats, compared with exosomes derived from BMCs (Barile et al., 2018). With further research, exosome miRNAs derived from CDCs or CPCs play an important role in myocardial protection. For example, Milano et al. (2020) showed that exosomes derived from CPCs miR-146a-5p can effectively alleviate doxorubicin (Dox)/trastuzumab (Trz)-induced oxidative stress in cardiomyocytes. Ibrahim et al. (2014) showed that miR-146a derived from CDCs exosomes can reduce scar formation after myocardial infarction in rats, inhibit cardiomyocyte apoptosis, and improve heart function. Therefore, further in-depth study of exosomal miRNA derived from CDCs or CPCs provides a new direction for cardioprotective therapy.

### Heart Failure and Exosomal miRNAs

Heart failure is caused by myocardial contraction dysfunction brought about by various factors. It leads to a decrease in cardiac blood output and blood stasis in the systemic or pulmonary circulation. HF is a major public health burden globally. Myocardial apoptosis, autophagy, inflammation, and myocardial cell remodeling are all involved in HF. Ventricular remodeling refers to continuous myocardial injury, excessive mechanical load, and other factors that lead to the overexpression of inflammatory cytokines and overactivation of the neuroendocrine system, resulting in changes in the myocardial cell structure and function. The main pathological process of ventricular remodeling is myocardial cell thickening, ventricular volume enlargement, and cardiac cavity shape change, with the cardiac function changing from compensatory to decompensatory, which eventually leads to HF.

According to recent studies, exosomal miRNAs play an important role in myocardial remodeling (Danielson et al., 2018). Wang et al. (2018) reported altered levels of serum exosomal miR-21, miR-425, and miR-744 in 31 patients with HF. The authors also observed down-regulation of miR-425 and miR-744 levels in CFs treated with angiotensin. The decrease in miR-425 and miR-744 levels was associated with the increase in collagen type 1 and α-SMA expression, which led to the activation of CFs. Further analysis indicated that miR-425 and miR-744 inhibit the synthesis of collagen and cellulose induced by angiotensin, and also inhibit myocardial remodeling, by targeting TGF-β. In addition, the miR-425 and miR-744 levels are altered in the serum of patients with HF; therefore, miR-425 and miR-744 are expected to be therapeutic targets for reversing myocardial remolding and markers for diagnosing HF.

Wu et al. (2018) observed that serum exosomal miRNA can be used as a biomarker of HF with reduced ejection fraction. The authors showed that the exosomal miR-92b-5p levels are increased in the serum of patients with acute HF, which was negatively correlated with the left ventricular ejection fraction. Therefore, the serum levels of exosomal miR-92b-5p can be used as a biomarker for diagnosing HF with a reduced ejection fraction. In summary, exosomal miRNAs are expected to become a novel tool for diagnosing and treating HF.

### Pulmonary Arterial Hypertension (PAH) and Exosomal miRNAs

Pulmonary arterial hypertension is a rare disease characterized by remodeling of the distal pulmonary circulation vessels. Vascular remodeling caused by pulmonary hypertension includes proliferation and apoptosis of the pulmonary vascular endothelial cells and extracellular matrix deposition, which leads to the loss of peripheral arterioles and occlusion of residual vascular beds. With the progression of PAH, pulmonary vascular resistance increases, resulting in right HF and death. Currently, some drugs are available that show certain selectivity for pulmonary vascular dilatation. These drugs improve pulmonary vascular resistance to a certain extent and delay the deterioration of PAH patients’ condition (Mathew, 2014; Rabinovitch et al., 2014). However, it is likely that curing PAH will require a drug that reverses pulmonary vascular remodeling caused by pulmonary hypertension.

It is not clear how pulmonary hypertension causes pulmonary vascular remodeling. Altered metabolism of endogenous pulmonary vasodilators, growth factors, and inflammatory pathways in PAH has been confirmed, but it is not clear whether these abnormalities also lead to changes in cell signaling.

Exosomal miRNAs play an important role in reversing pulmonary hypertension (Klinger et al., 2020). In cardiovascular diseases, the miR-143/145 cluster is the best studied miRNA cluster. Indeed, miR-145 and miR-143 are widely involved in cardiovascular diseases, such as atherosclerosis, coronary heart disease, aortic dissection, valvular disease, and pulmonary hypertension (Caruso et al., 2012; Liu et al., 2013, 2019; Guo et al., 2016; Girdauskas et al., 2018; Shi et al., 2018; Sun et al., 2018; Yue et al., 2018; Li T. et al., 2019; Vacante et al., 2019). Deng et al. (2015) reported that exosomal miR-143 regulates the interaction between VSMCs and endothelial cells in PAH. The authors observed that miR-143 levels are significantly increased in pulmonary vascular tissue in an animal model of pulmonary hypertension. Deletion of the miR-143 gene in mouse prevented the development of PAH induced by hypoxia. In addition, activation of the miR-143/145 gene cluster involves an upstream promoter region, which includes many binding elements; the cluster is activated by PAH-associated signaling pathways. MiR-143 regulates migration and apoptosis of pulmonary artery smooth muscle cells (PASMCs). Exosomal miR-143 derived from PASMCs promotes the migration and angiogenesis of endothelial cells. This highlights the intercellular communication between PASMCs and pulmonary artery endothelial cells (PAECs) via exosomal miR-143 during the progression of pulmonary hypertension, indicating that miR-143 is an important regulator of PAH. Consequently, a treatment strategy targeting miR-143 may constitute a highly beneficial treatment for this disease. In addition, Sindi et al. (2020) demonstrated that exosomal miR-181a-5p and miR-324-5p reverse pulmonary vascular remodeling caused by PAH. Based on these observations, exosomal miRNA might be a new therapeutic target for reversing vascular remodeling caused by PAH.

### Aortic Aneurysm and Exosomal miRNA

Aortic aneurysm refers to an aneurysm-like expansion of the aorta, usually an increase of the diameter by more than 50%. The most serious complication of aortic aneurysm is rupture of the aorta, which is life-threatening. Smoking, advanced age, and male sex are strong risk factors for the development of aortic aneurysm. Indeed, among individuals over 55 years old, the incidence of abdominal aortic aneurysm (AAA) is 4–7% among males and 1–2% among females (Golledge et al., 2006; Moll et al., 2011; Sidloff et al., 2014). At present, the diagnosis of AAA involves monitoring the diameter of the aorta. The current treatment methods are open surgery and endovascular repair. However, to date, no clinical trials of drugs that could limit the progression or rupture of AAA have been reported. Therefore, exploring the mechanism of AAA initiation and progression may be a new target to limit the AAA progression or rupture.

As in other cardiovascular disease, exosomal miRNAs are involved in AAA. For example, Han et al. (2020) showed that, compared with healthy individuals, exosomal miR-106a levels are elevated in AAA patients. Further analysis revealed that exosomal miR-106a promotes apoptosis of VSMCs. In addition, exosomal miR-106a increases the levels of matrix metalloproteinases (including matrix metalloproteinases 2 and 12) secreted by VSMCs via a targeted regulation of TIMP-2, which promotes the progression of AAA.

Maegdefessel et al. (2014) reported that miR-24 is the key regulator of vascular inflammation and AAA pathology in a mouse AAA disease model, and human aortic tissue and plasma. MiR-24 regulates the synthesis of cytokines in M1 macrophages by targeting CHI3L1, promotes the migration of VSMCs, and stimulates the expression of vascular endothelial adhesion molecules. The authors demonstrated that regulation of miR-24 levels alters the progression of AAA in animal models, and that miR-24 represents a new plasma biomarker of human AAA disease progression. Collectively, exosomes could become a new target for reversing the expansion of aortic aneurysm.

### Vascular Calcification (VC) and Exosomal miRNA

Vascular calcification is a pathological calcification of blood vessels, caused by abnormal metabolism of calcium and phosphate, osteogenic differentiation, inflammation, and other factors. It is closely related to the occurrence of major adverse cardiovascular events (MACEs). VC usually occurs under the intima of the blood vessel wall. A large number of studies published recently have demonstrated that VC is closely associated with the atherosclerotic plaque rupture. According to some clinical studies, MACE is more likely to occur during moderate or severe calcification during coronary vascularization than during non/mild calcification. Therefore, reversing the therapeutic target of VC could be a feasible method for reducing the occurrence of MACE. Statins are the most widely used representative drugs for VC treatment. However, although statins can effectively reduce the levels of low-density lipoprotein, they cannot alleviate the process of coronary artery calcification. Therefore, identification of a new target for reversing VC therapy is urgently needed.

Exosomal miRNAs play an important role in the formation of VC. For example, Gui et al. (2012) showed that elevated levels of miR-135, miR-762, miR-714, and miR-712 in VSMCs participate in VC by interfering with calcium efflux protein. Further, Xia et al. (2015) showed that the Runx2/miR-3960/miR-2861 positive feedback loop is responsible for the transformation of VSMCs into osteoblasts and promotes the formation of VC. It has been also reported that exosomal miRNAs are involved in the formation of VC (Kapustin and Shanahan, 2016; Liberman and Marti, 2017; Zhang C. et al., 2018). For example, Pan et al. (2020) established a calcification model in the rat smooth muscle and MOVAS-1 cell line. The exosomal let-7e-5p levels decreased and exosomal miR-324-3p levels increased in the MOVAS-1 cell calcification model. Further analysis indicated that miR-324-3p regulates MOVAS-1 calcification via IGF1R, PI3KCA, and MAP2K1. Therefore, exosomes may be developed into a new-generation targeted drugs to prevent VC and promote the stability of atherosclerotic plaques.

### Rheumatic Valvular Disease (RVD) and Exosomal miRNA

Rheumatic heart disease (RHD) is a major problem in developing countries and the main cause of cardiovascular death among young people. It is an autoimmune disease caused by an abnormal antigen cross-reaction between group A streptococci and human connective tissue. RVD refers to the abnormal function of the valve and hemodynamic disturbances caused by repeated episodes of rheumatic carditis involving the heart valve and its accessory structures. Severe valve deformity results in high mortality. RVD most often involves the mitral valve, followed by the aortic valve, the latter often with the mitral valve damaged at the same time, which is known as the combined valvular disease (Chopra and Gulwani, 2007; Marijon et al., 2012; Liu et al., 2015). According to recent studies, the S1PR1/STAT3 signaling pathway participates in RHD-induced cardiac valve injury by regulating Th17 cells (Wu et al., 2019). Further studies revealed that exosomal miRNA is involved in the regulation of this pathway during the development of RHD. Chen et al. (2020) observed that the serum levels of exosomal miR-155-5p are closely related to valve injury. Indeed, the exosomal miR-155-5p levels are increased in an RHD rat model. Further analysis revealed that exosomal miR-155-5p enhances the expression of S1PR1 and inhibits the activation of the SOCS1/STAT3 signaling pathway, thereby reducing the degree of valve inflammation and fibrosis, and reducing the levels of IL-6 and IL-17 in the valve tissue and serum. These observations suggest that inhibition of miR-155-5p can reduce RHD-induced valve impairment through the S1PR1, SOCS1/STAT3, and IL-6/STAT3 signaling pathways. Although the role of exosomal miRNA in RVD has not been extensively studies, exosomal miRNA is nonetheless a promising and important tool for the treatment and diagnosis of RVD.

## Conclusion

**FIGURE 3:**
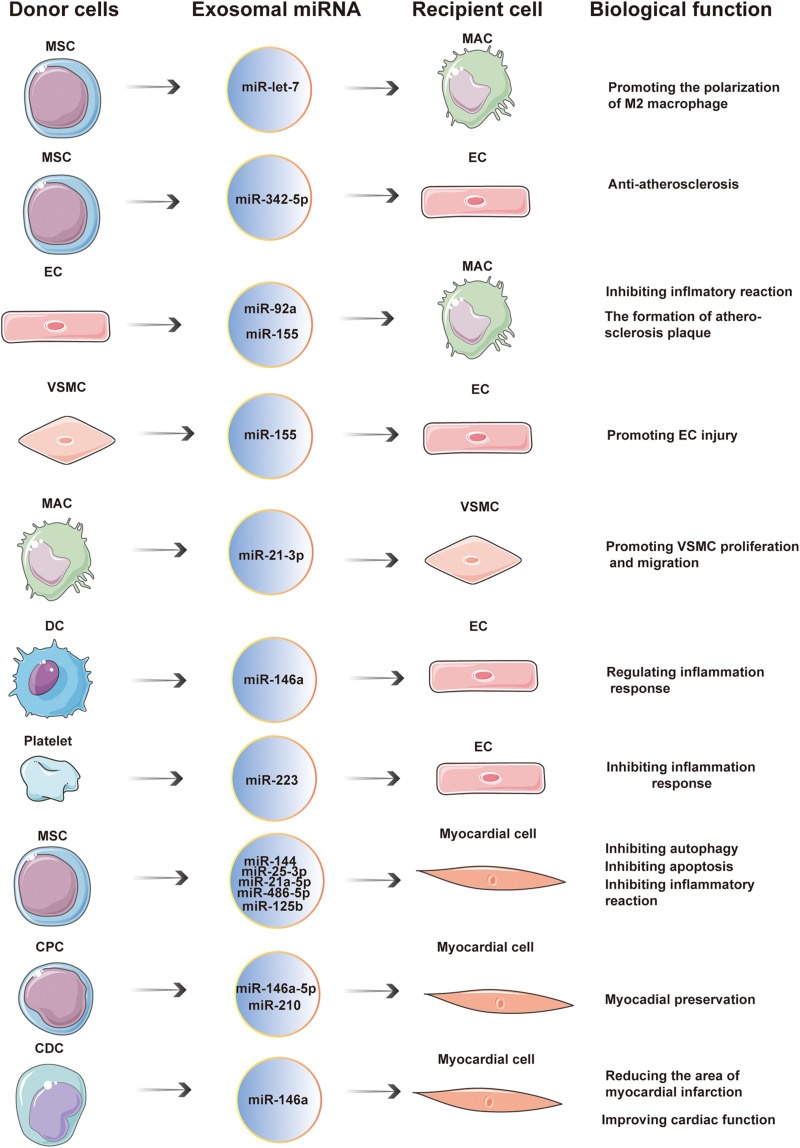
Exosomal miRNAs are widely involved in cardiovascular diseases. In the occurrence and development of cardiovascular diseases, exosomal miRNA is an important way of communication between cells. Donor cells secrete exosomal miRNAs which are taken up by recipient cells. The entry of exosomal miRNA into recipient cells exerts a wide range of biological effects through post-transcriptional regulation.

In conclusion, exosomal miRNAs play an important role in cardiovascular diseases. These molecules are widely involved in the occurrence and development of cardiovascular diseases, such as atherosclerosis, ACS, HF, myocardial infarction, and pulmonary hypertension. Exosomal miRNAs are an important means of intercellular communication. As such, they can impede atherosclerosis, reduce MIRI, improve myocardial function after HF, and regulate the inflammatory response (Figure 3 and Table 1). In addition, some exosomal miRNAs are differently expressed in the serum of patients with atherosclerosis, HF, ACS, and other cardiovascular diseases, compared with healthy patients, and are closely related to the diagnosis and severity of these diseases (Table 2). Therefore, exosomal miRNAs are expected to be developed into a novel tool for diagnosing and treatment of cardiovascular diseases.

**TABLE 1:** The role of exosomal miRNA in cardiovascular disease.

miRNA	Donor cell	Recipient cell	Pathway	Disease	Function	References
miR-let7	MSCs	MACs	IGFZBP1/PTEN HMGA2/NF-κB	AS	Promoting the polarization of M2 macrophage	Li J. et al. (2019)
miR-342-5p	MSCs	ECs	PPP1R12B	AS	Anti-atherosclerosis	Xing et al. (2020)
miR-155	ECs	MACs	KLF2/miR-155	AS	Inhibiting inflammatory reaction	He et al. (2018)
miR-92a	ECs	MACs	Targeting KLF4	AS	The formation of atherosclerotic plaque	Loyer et al. (2014), Chang et al. (2019)
miR-155	VSMCs	ECs	KLF5/miR-155	AS	Promoting endothelial cell injury	Zheng et al. (2017)
miR-21-3p	MACs	VSMCs	PTEN	AS	Promoting VSMCs proliferation and migration	Zhu et al. (2019)
miR-106a-3p	MACs	VSMCs	N/A	AS	Promoting VSMCs proliferation and inhibiting VSMCs apoptosis	Liu et al. (2020)
miR-146a	DCs	ECs	Targeting IRAK-1	AS	Regulating inflammation response	Zhong et al. (2019)
miR-223	Platelets	ECs	miR-223/ICAM-1	AS	Inhibiting inflammatory reaction	Li et al. (2017)
miR-125b	MSCs	Myocardial cells	Targeting SIRT7	AMI	Reducing the area of myocardial infarction	Zhu et al. (2018)
miR-21a-5p	MSCs	Myocardial cells	PDCD4, PTEN, Peli1, FasL	AMI	Myocardial protection	Wang et al. (2017), Luther et al. (2018)
miR-301	MSCs	Myocardial cells	N/A	AMI	Inhibiting autophagy of myocardial cells	Li Y. et al. (2019)
miR-25-3p	MSCs	Myocardial cells	Targeting E2Z2	AMI	Reducing the area of myocardial infarction	Peng et al. (2020)
miR-144	MSCs	Myocardial cells	PTEN/AKT	AMI	Improving the apoptosis of cardiomyocytes under hypoxia	Wen et al. (2020)
miR-126	N/A	Myocardial cells	Targeting ERRHI1	AMI	Improving myocardial cell survival rate	Wang et al. (2019)
miR-423-3p	Cardiac fibroblasts cells	Myocardial cells	Targeting RAP2C	MIRI	Reducing myocardial cells apoptosis	Luo et al. (2019)
miR-486-5p	MSCs	Myocardial cells	PTEN/PI3K/Akt	MIRI	Repairing myocardial injury	Sun et al. (2019)
miR-182	MSCs	MACs	Targeting TLR4	MIRI	Inhibiting inflammatory reaction	Zhao et al. (2019)
miR-143	VSMCs	ECs	N/A	PAH	Promoting ECs migration and angiogenesis	Deng et al. (2015)
miR-181a-5p	ECs	VSMCs	Norch4, ETS1	PAH	Reversing vascular remodeling	Sindi et al. (2020)
miR-324-5p	ECs	VSMCs	Norch4, ETS1	PAH	Reversing vascular remodeling	Sindi et al. (2020)
miR-106a	N/A	VSMCs	Targeting TIMP-2	AAA	Promoting VSMCs apoptosis	Han et al. (2020)
miR-24	N/A	MACs	Targeting CHI3L1	AAA	Regulating inflammation response	Maegdefessel et al. (2014)
let-7e-5p	N/A	VSMCs	IGF1R, PI3KCA and MAP2K1	VC	Regulating vascular calcification	Pan et al. (2020)
miR-324-3p	N/A	VSMCs	IGF1R, PI3KCA and MAP2K1	VC	Regulating vascular calcification	Pan et al. (2020)
miR-155-5p	N/A	N/A	S1PR1, SOCS1/STAT3	RVD	Regulating inflammation and fibrosis	Chen et al. (2020)

**TABLE 2:** The role of serum exosomal miRNAs in cardiovascular disease.

miRNA	Expression	Disease	Function	References
miR-208a	↑	ACS	Diagnostic and prognostic	Bi et al. (2015)
miR-126	↑	ACS	Diagnostic	Ling et al. (2020b)
miR-21	↑	ACS	Diagnostic	Ling et al. (2020b)
miR-143	↓	AMI	Therapeutic	Geng et al. (2020)
miR-425	↓	HF	Diagnostic and therapeutic	Wang et al. (2018)
miR-744	↓	HF	Diagnostic and therapeutic	Wang et al. (2018)
miR-92b-5p	↑	HF	Diagnostic	Wu et al. (2018)

*(↑) and (↓) indicate increased and decreased expression of miRNA, respectively.*

## Author Contributions

DZ and KL researched the article and wrote the manuscript. MH, BL, WW, HP, YW, ZZ, DL, and TW reviewed and edited the manuscript before submission. All authors provided substantial contribution to the discussion of content.

## Conflict of Interest

The authors declare that the research was conducted in the absence of any commercial or financial relationships that could be construed as a potential conflict of interest.

## Abbreviations

AAA: abdominal aortic aneurysm
ACS: acute coronary syndrome
AGO: argonaute protein
AMI: acute myocardial infarction
BMSC: bone marrow mesenchymal stem cell
CF: cardiac fibroblast
CPC: cardiac progenitor cells
CDC: cardiophere-derived cells
DGCR8: DiGeorge Syndrome critical region 8 homolog
ESCRT: endosomal-sorting complex required for transport
GW182: glycine-tryptophan protein of 182 Da
HF: heart failure
hnRNP: heterogeneous nuclear ribonucleoprotein
H/R: hypoxia and reoxygenation
ILV: intraluminal vesicle
lncRNA: long non-coding RNA
MACE: major adverse cardiovascular event
MAC: macrophage
MHC: major histocompatibility complex
MIRI: myocardial ischemia-reperfusion injury
miRNA: microRNA
MSC: mesenchymal stem cell
MVB: multivesicular body
PAH: pulmonary arterial hypertension
PASMC: pulmonary artery smooth muscle cells
pri-miRNA: primary precursor of miRNAs
RHD: rheumatic heart disease
RISC: RNA-induced silencing complex
ROC: receiver operating characteristic
RVD: rheumatic valvular disease
SNARE: soluble *N*-ethylmaleimide-sensitive factor attachment protein receptor
TAA: thoracic aortic aneurysm
TLR: toll-like receptor
UA: unstable angina pectoris
VC: vascular calcification
VSMC: vascular smooth muscle cell.
